# Effect of Luting Cement on Marginal and Internal Adaptation of Novel Ceramic-Reinforced Polymer Crowns: A Micro-CT Study

**DOI:** 10.3390/polym18141714

**Published:** 2026-07-13

**Authors:** Naluemol Sriprasert, Nantawan Krajangta, Thanakorn Wasanapiarnpong, Pavinee Padipatvuthikul Didron, Thanasak Rakmanee

**Affiliations:** 1 Department of Restorative and Esthetic Dentistry, Faculty of Dentistry, Thammasat University, Patum Thani 12120, Thailand; naluemol.s@rsu.ac.th (N.S.); knantawa@tu.ac.th (N.K.); 2 College of Dental Medicine, Rangsit University, Patum Thani 12000, Thailand; 3 Upcycled Materials from Industrial and Agricultural Wastes Research Unit, Department of Materials Science, Faculty of Science, Chulalongkorn University, Bangkok 10330, Thailand; thanakorn.w@chula.ac.th; 4 Department of General Dentistry, Faculty of Dentistry, Srinakharinwirot University, Bangkok 10110, Thailand; pavinee.didron@gmail.com

**Keywords:** ceramic-reinforced polymer, micro-CT, marginal gap, absolute marginal discrepancy, internal gap

## Abstract

A novel alumina-filled ceramic-reinforced polymer (CRP) crown (Hassawat-01; HS) was developed. This study evaluated the effect of luting cement on the marginal and internal adaptation of HS and compared its performance with a commercial DLP-printed CRP (VarseoSmile Crown Plus^®^; VS) and a milled resin nanoceramic (Cerasmart^®^ 270; CE). Ninety-nine crowns (n = 33/material) were fabricated with a 50 µm cement space and luted using Maxcem Elite^®^, RelyX Unicem^®^, or Ketac Cem^®^ (n = 11/subgroup). Adaptation was assessed without and with cementation using micro-computed tomography at 160 measurement points per crown. Without cementation, HS demonstrated the most favorable internal adaptation, whereas VS showed the best marginal adaptation. Following cementation, gap dimensions increased in all groups. Despite its superior non-cementation fit, HS exhibited the greatest increase in marginal and internal discrepancies, suggesting increased hydraulic resistance during seating. Among the evaluated cement–crown combinations, VS luted with RelyX Unicem^®^ showed the most favorable post-cementation adaptation. Post-cementation analysis was limited to HS and VS because the radiopacity of CE prevented reliable cement interface segmentation. These findings indicate that adaptation is influenced by both crown geometry and cement properties, and that highly adapted intaglio surfaces may require careful cement selection to optimize clinical fit.

## 1. Introduction

Ceramic-reinforced polymers (CRPs) are CAD/CAM-compatible hybrid restorative materials developed to integrate the favorable mechanical performance of ceramics with the resilience and processability of polymer matrices. Structurally, CRPs consist of a cross-linked organic polymer network reinforced by a dispersed ceramic phase and are typically manufactured under high temperature and pressure to enhance monomer conversion and cross-link density, thereby improving mechanical stability and interfacial bonding [1,2,3]. CRPs are broadly classified into polymer-infiltrated ceramic networks (PICNs) and resin nanoceramics (RNCs). PICNs comprise an interconnected glass–ceramic scaffold infiltrated with polymer, forming a three-dimensional interpenetrating network [1], whereas RNCs contain silica nanoparticles and zirconia or barium glass fillers dispersed within a highly cross-linked organic resin matrix [4,5]. Among commercially available RNCs, Cerasmart^®^ 270 (CE), containing approximately 71 wt.% ceramic fillers (20 nm silica and 300 nm barium glass), has demonstrated favorable clinical performance [3].

Typically, CRP restorations are fabricated by subtractive CAD/CAM milling of pre-polymerized blocks. Although milling ensures industrially controlled polymerization and consistent material properties, it results in material waste and may introduce surface or subsurface microcracks. Additive manufacturing, particularly digital light processing (DLP), has emerged as an alternative fabrication strategy offering improved material efficiency, enhanced polymerization control, and potentially more homogeneous filler distribution [1,6]. However, DLP-printable CRPs usually require reduced filler loading to maintain adequate flowability and light penetration during printing, which may compromise mechanical strength and long-term dimensional stability [7,8,9]. For example, VarseoSmile Crown Plus^®^ (VS), a DLP-printable CRP containing 30–50 wt.% silanized ceramic fillers, is indicated for single-unit restoration; nevertheless, its relatively lower filler content compared with milled RNC blocks has raised concerns regarding long-term mechanical stability [7,8,9].

To overcome the filler loading limitations of DLP-printable CRPs, a novel material, Hassawat-01 (HS), was developed using alumina as the primary reinforcing phase within a urethane acrylate (UA)-based polymer matrix. Alumina enhances mechanical strength, wear resistance, and structural stability without undesirable interfacial reactions, and has been shown to improve fracture toughness and crack resistance through crack-deflection and crack-pinning mechanisms at the filler–matrix interface [10,11]. Although excessive filler loading may reduce flexural strength due to limited crack-arresting capability [11], the UA-based matrix was selected for its high toughness and strong cross-linking capability, as well as its low polymerization shrinkage (0.81–0.91%) and high double bond conversion during DLP fabrication, which may contribute to improved dimensional fidelity of the printed restorations [12,13,14]. To balance printability and mechanical performance, HS was formulated with 38.5 wt.% of 0.7 µm alumina fillers and 61.5 wt.% UA-based resin [15]. Although HS has previously demonstrated favorable fracture resistance [15], fracture resistance and marginal/internal adaptation represent different clinical outcomes governed by different mechanisms. To date, the three-dimensional adaptation of HS following cementation has not been quantitatively investigated. Therefore, the present study specifically addresses this knowledge gap using quantitative micro-CT analysis.

Marginal and internal adaptation are critical determinants of the long-term clinical success of indirect restorations. Inadequate fit may contribute to microleakage, secondary caries, pulpal or periodontal inflammation, and loss of retention [16]. Clinically acceptable marginal gaps have been traditionally proposed to be below 120 µm [17], with many studies reporting acceptable values between 25 and 120 µm, although discrepancies up to 200 µm may be tolerated under certain conditions [17,18,19,20]. Reported marginal gaps for CRP restorations range from 33.9 to 407.5 µm depending on fabrication technique, material composition, and measurement methodology [18,21,22,23,24]. Although DLP-printed CRP crowns have generally demonstrated favorable adaptation compared with milled counterparts in pre-cementation evaluations, results are not uniform across studies, and adaptation remains highly dependent on resin composition, printing parameters, and post-curing protocols [25,26].

Final clinical adaptation is not determined solely by fabrication accuracy, as cementation can substantially alter seating dynamics. Luting cement introduces hydrodynamic resistance during seating, increases cement film thickness, and may compromise marginal integrity, resulting in post-cementation gap increases that have been consistently reported regardless of crown material or cement type [27,28,29,30,31]. In feldspathic ceramic and zirconia restorations, post-cementation marginal gap increases of approximately 28–70 µm have been attributed to cement viscosity, polymerization behavior, internal relief design, and intracoronal hydraulic pressure [27,28,29,30,31]. Although increasing cement space may facilitate seating and reduce marginal discrepancies, excessive spacing may negatively affect internal adaptation. Within digital CAD/CAM workflows, cement space can be customized similarly to conventional die spacers, yet no consensus currently exists regarding optimal settings, with reported values ranging from 0 to 90 µm depending on the system, restoration type, and material [32,33,34,35,36,37,38,39]. The type of luting cement may also influence marginal integrity. Self-adhesive resin cements, such as Maxcem Elite^®^ and RelyX Unicem^®^, simplify clinical procedures by eliminating separate etching and priming steps [32], yet studies have reported inconsistent results regarding marginal sealing performance [40,41,42]. Glass-ionomer cements (GICs), such as Ketac Cem^®^, offer advantages including low film thickness, hydrophilicity, and fluoride release [43]; nevertheless, their behavior in DLP-printed CRP crowns remains insufficiently characterized.

Assessment of restoration fit is also influenced by measurement methodology. Conventional techniques, such as sectioning and silicone replica methods, present inherent limitations: sectioning is destructive and prevents repeated measurements, whereas the silicone replica technique is susceptible to distortion of ultrathin layers [44,45]. Micro-computed tomography (micro-CT) enables high-resolution, non-destructive three-dimensional evaluation across multiple planes and has been widely applied to assess crown fit. Marginal adaptation is typically quantified by absolute marginal discrepancy (AMD) and marginal gap (MG), while internal adaptation is assessed at predefined anatomical locations including the finish line, axial wall, and occlusal surface. Although micro-CT requires higher cost and longer processing time, it provides standardized and reproducible assessment that is particularly suited for pre- and post-cementation comparisons [46,47,48,49].

Among studies most directly relevant to the present investigation, Suksuphan et al. [24] evaluated pre-cementation marginal adaptation and fracture resistance of milled CE, PICN, and DLP-printed VS crowns, reporting superior marginal adaptation in DLP-printed crowns; however, adaptation was assessed only before cementation and using a single cement type. Khwanpuang et al. [1] demonstrated that printing orientation significantly affected internal and external gap distribution of DLP-printed hybrid ceramic crowns, highlighting the importance of fabrication parameters on adaptation outcomes. Kakinuma et al. [21] reported superior trueness and fewer marginal discrepancies in DLP-printed resin-composite crowns compared with milled counterparts, particularly in complex internal geometries; nonetheless, post-cementation adaptation was not evaluated. Collectively, these studies did not address the influence of cement type on post-cementation adaptation of DLP-printed CRP crowns. More importantly, the marginal and internal adaptation of HS—a novel alumina-reinforced DLP-printable CRP developed as part of the present research program under clinically relevant cementation conditions has not been characterized.

Accordingly, this study evaluated the marginal and internal adaptation of three CAD/CAM-based CRP crown materials (HS, VS, and CE) without cementation and the post-cementation adaptation of DLP-printed materials (HS and VS) using micro-CT. The influence of three luting cements (Maxcem Elite^®^, RelyX Unicem^®^, and Ketac Cem^®^) was also investigated. The null hypotheses were that (1) without cementation, marginal and internal adaptation would not differ among the three CRP materials; (2) among the DLP-printed CRP materials (HS and VS), luting cement type would not affect marginal or internal adaptation after cementation; and (3) there would be no interaction between crown material and luting cement type on post-cementation marginal or internal adaptation.

## 2. Materials and Methods

### 2.1. Study Design

The same specimen cohort used in our previous study [15] was employed; however, the present investigation addressed a different primary outcome by quantitatively evaluating three-dimensional marginal and internal adaptation without and with cementation. A total of 99 CAD/CAM-fabricated mandibular first molar crowns were evaluated. Three ceramic-reinforced polymer (CRP) materials with different fabrication techniques were included: an experimental alumina-reinforced DLP-printed CRP (Hassawat-01; HS), a commercial DLP-printed CRP (VarseoSmile Crown Plus^®^; VS), and a milled resin nanoceramic (Cerasmart^®^ 270; CE), with 33 specimens per material. HS and VS crowns were fabricated using a DLP-printing system (Freeform Pro 2; ASIGA, Sydney, NSW, Australia) from standardized STL files of a unified digital crown design [24], whereas CE crowns were milled using a CARES M Series system (Straumann, Basel, Switzerland). Printed specimens were ultrasonically cleaned in 96% ethanol, air-dried, and post-cured according to manufacturer protocols to ensure complete polymerization [50].

Sample size determination was performed using G*Power software (version 3.1; Heinrich-Heine-Universität Düsseldorf, Germany) with a significance level of α = 0.05 and statistical power of 0.80. The effect size was derived from a previously published study evaluating the marginal adaptation of CAD/CAM crowns and fracture resistance [24]. The analysis indicated that a minimum of 11 specimens per group was required to detect statistically significant differences in marginal discrepancy.

Prior to cementation, a randomly selected subset of 11 crowns from each material group (HS, VS, and CE) and resin dies was scanned using high-resolution micro-computed tomography (micro-CT) (SkyScan 1172, Bruker micro-CT, Kontich, Belgium) to obtain baseline non-cementation adaptation data. Marginal and internal adaptation were evaluated using micro-CT at 160 predefined measurement points per crown. Marginal adaptation was quantified using absolute marginal discrepancy (AMD) and marginal gap (MG), whereas internal adaptation was assessed at the shoulder area (SA), axial space (AS), and occlusal space (OS). As micro-CT is a non-destructive technique, these specimens were subsequently returned to their respective groups.

**Figure 1:**
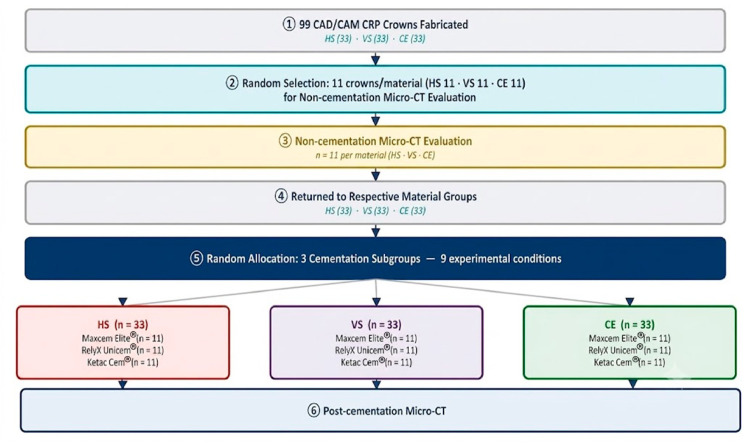
Conceptual framework of the study. HS = Hassawat-01; VS = VarseoSmile Crown Plus^®^; CE = Cerasmart^®^ 270. n = number of specimens per group.

Each material group (n = 33) was then randomly allocated into three luting cement subgroups (n = 11): Maxcem Elite^®^ (~4 GPa), RelyX Unicem^®^ (~13 GPa), and Ketac Cem^®^ (~20 GPa), resulting in nine experimental conditions. Before luting, the intaglio surfaces of all crowns were airborne-particle abraded with 50 µm aluminum oxide (1.5 bar, 10 s, 10 mm distance) and treated with a silane coupling agent (RelyX Ceramic Primer^®^, 3M ESPE, St. Paul, MN, USA) [7,51]. The crowns were then luted with the respective cement to standardized 3D-printed resin dies (Rigid 10K; Formlabs Inc., Somerville, MA, USA) and stored at 37 °C for 24 h. The same micro-CT measurement protocol was subsequently repeated to evaluate post-cementation marginal and internal adaptation. The study design and material compositions are summarized in Table 1 and Figure 1, respectively.

### 2.2. Resin Dies: Design and Preparation

A resin die representing a mandibular first molar was designed using Autodesk Fusion 360 (version 2.0.16786; Autodesk Inc., San Francisco, CA, USA) and exported as an STL file. The preparation followed standardized full-coverage crown principles [15], incorporating 1.0 mm occlusal reduction, 1.2 mm proximal and axial reduction, a convergence angle of 6° per axial wall, and a 0.8 mm chamfer finish line with rounded internal line angles. A total of 99 identical resin dies were fabricated using a Form 3B stereolithography (SLA) 3D printer (Formlabs Inc., Somerville, MA, USA) with a high-strength, glass-filled photopolymer resin (Rigid 10K; Formlabs Inc., Somerville, MA, USA). Post-curing was performed in a Form Cure unit (Formlabs Inc., Somerville, MA, USA) at 70 °C for 60 min according to the manufacturer’s instructions to ensure complete polymerization. The fabricated dies were stored in a light-protected environment at room temperature until crown fabrication.

### 2.3. CRP Crown Specimens: Digital Design, Fabrication and Cementation

#### 2.3.1. Digital Design

A standardized CRP crown was designed using 3Shape Dental System (Version 2020). The virtual design incorporated a uniform occlusal thickness of 1.0 mm and a cement setting of 50 µm between the intaglio surface of the crown and the resin die.

#### 2.3.2. Fabrication CRP Crown Specimens

For the additive groups, 33 HS and 33 VS crowns were produced using a DLP 3D printer (Freeform Pro 2; ASIGA, Sydney, NSW, Australia). STL files were processed using ASIGA Composer (version 2.1.0; ASIGA, Sydney, NSW, Australia) and printing was performed according to the manufacturer’s recommended parameters (50 µm layer thickness, 12.816 s exposure time, and 7.6 mW/cm^2^ light intensity). For the subtractive group, 33 CE crowns were milled using a CARES M Series system (Straumann, Basel, Switzerland) with 1.0-, 1.4-, and 1.8 mm burs, replaced after every 17 crowns to maintain machining accuracy.

Printed crowns underwent standardized post-processing: airborne-particle abrasion with 50 µm aluminum oxide (1.5 bar, 10 s, 10 mm distance), ultrasonic cleaning in 96% ethanol, and air-drying. Post-curing was performed under nitrogen using a BEGO Otoflash unit (BEGO, Bremen, Germany; 300–700 nm, 10 Hz) for two cycles of 1500 flashes, with a 5 min cooling interval between cycles [50]. The experimental HS material was processed using the same equipment, software workflow, and post-curing protocol as the commercial VS material to ensure methodological consistency.

#### 2.3.3. Cementation

Prior to luting, the intaglio surfaces were treated as described above. To ensure consistent seating and monitor potential crown displacement, reference markings were placed on both the crown and the die at four locations. Complete seating was verified by confirming alignment of these markings before and after cementation.

For the resin cements (Maxcem Elite^®^ and RelyX Unicem^®^), a 20 N seating load was applied using a digital force gauge (SF-500; Wenzhou Sanhe, Wenzhou, China) mounted on a manual screw stand, and maintained throughout the initial cementation steps. Following verification of complete seating, the cement was tack-cured for 2 s per axial surface, and gross excess cement was removed with a probe [52]. Each axial surface was then light-cured for 20 s while the seating load remained applied. The seating device was subsequently removed, and each axial surface received an additional 20 s of light curing using an LED unit (470 nm, 1000 mW/cm^2^; SmartLite Focus, Dentsply Sirona, Charlotte, NC, USA). Final curing was then performed on the occlusal surface for 40 s.

For Ketac Cem^®^, the 20 N seating load was maintained for 7 min, according to the manufacturer’s instructions [24,53,54]. Excess cement was removed with a probe at approximately 7 min from the start of mixing, at the initial setting stage [55].

All specimens were stored at 37 °C for 24 h prior to micro-CT analysis.

### 2.4. Micro-CT Analysis of Adaptation

Marginal and internal adaptation were evaluated using micro-CT system with the following acquisition parameters: 67 kV tube voltage, 41 µA tube current, 10 µm isotropic voxel size, and a 1 mm aluminum filter. Specimens were positioned perpendicular to the X-ray beam, and projection images were collected throughout a 360° rotation with a 0.2° rotation step. Projection images were reconstructed into cross-sectional slices using NRecon software (v1.6.9; Bruker), and the reconstructed datasets were used to visualize cement distribution and internal structural morphology using DataViewer software (v1.5.1.2; Bruker) [15].

**Figure 2:**
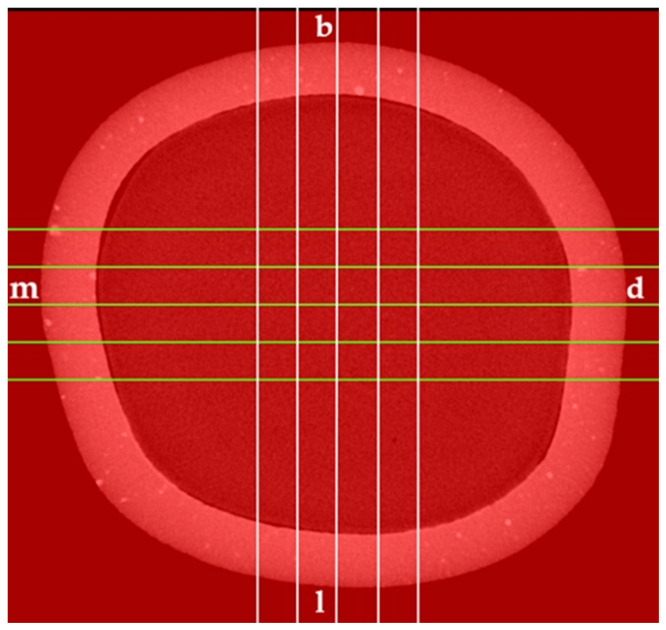
Representative micro-CT cross-sections. Ten cross-sectional slices were obtained per specimen by establishing central reference lines in both mesio-distal and bucco-lingual directions. White lines indicate the mesio-distal sections, and green lines indicate the bucco-lingual sections. Sections were generated at 100-slice intervals up to ±200 slices from the centerline, resulting in five equidistant sections in each direction (−200, −100, 0, +100, +200). b = buccal; l = lingual; m = mesial; d = distal.

**Figure 3:**
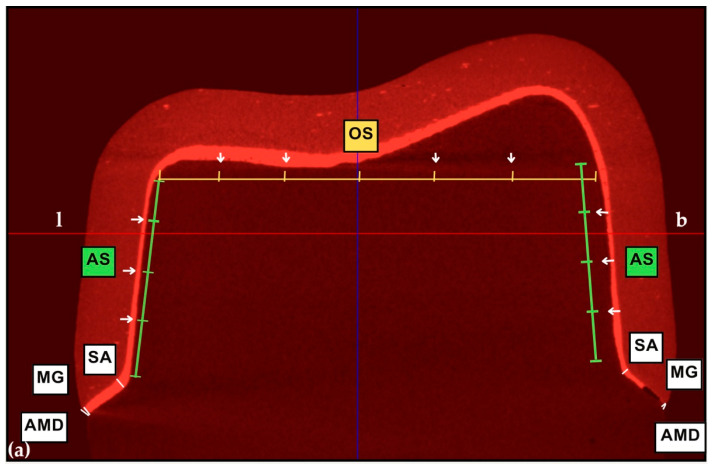

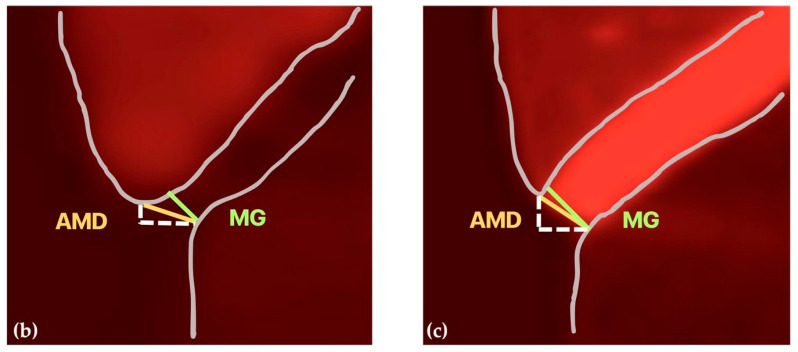
(**a**) Representative micro-CT cross-sectional image from a single slice illustrating the described absolute marginal discrepancy (AMD), marginal gap (MG), shoulder area (SA), axial space (AS), and occlusal space (OS). White arrows indicate the measurement locations. (**b**) Schematic illustration of AMD and MG before cementation and (**c**) after cementation. In panels (**b**,**c**), the yellow and green lines indicate the measurement locations for AMD and MG, respectively. b = buccal; l = lingual.

For standardized measurement, a systematic sectioning protocol was applied to each specimen (Figure 2). A central reference line was first established along the mesio-distal axis of the crown. Cross-sectional slices were then generated at 100-slice intervals symmetrically on both sides of this central line, extending to ±200 slices, yielding five equidistant mesio-distal sections at positions −200, −100, 0, +100, and +200 relative to the centerline. The identical procedure was subsequently applied in the bucco-lingual direction, generating five additional equidistant sections. In total, ten cross-sectional slices were obtained per specimen—five in the mesio-distal plane and five in the bucco-lingual plane. Within each of the ten cross-sectional slices, sixteen standardized measurement points were defined according to predefined anatomical reference regions: two points for absolute marginal discrepancy (AMD), two points for marginal gap (MG), two points for shoulder area (SA), six points for axial space (AS), and four points for occlusal space (OS).This 16-point measurement protocol was applied consistently and identically across all ten sections per specimen, yielding a total of 160 measurement points per crown, comprising 20 AMD, 20 MG, 20 SA, 60 AS, and 40 OS measurements (Figure 3).

AMD and MG were defined according to Holmes et al. [56]. Briefly, AMD represents the combined vertical and horizontal discrepancy at the crown margin, including over or under extension of the restoration, whereas MG is defined as the perpendicular distance between the internal crown surface and the preparation margin at the finish line. The SA was defined as the perpendicular internal gap at the transition between the finish line and the axial wall. Two SA points were recorded per section, one on each side of the crown. For AS, the axial wall extending from the SA region to the occlusal transition was divided into five equal segments on both sides of the crown. The three central reference points on each side were selected for measurement, resulting in six AS points per section. To standardize measurements, segmentation was initiated from the side with the shorter axial wall height. For OS, a horizontal reference line parallel to the occlusal plane was drawn from the highest point of the shorter axial wall to the opposing side. The line was divided into six equal segments, and four predefined points were used for OS measurements, resulting in four OS points per section.

All measurements were performed using DataViewer software under standardized magnification and viewing conditions. Two blinded and calibrated examiners independently performed all measurements. Intra- and inter-observer reliability were assessed using the intraclass correlation coefficient (ICC), calculated with a two-way random-effects model and absolute agreement (single measures). The intra-observer ICC was 0.94, while the inter-observer ICC was 0.92, indicating excellent reliability.

### 2.5. Statistical Analysis

Statistical analysis was performed using SPSS software (version 26.0; IBM Corp., Armonk, NY, USA). All analyses were based on crown-level mean values, whereby measurement points within each parameter were averaged to obtain a single representative value per crown. Data normality and homogeneity of variance were assessed using the Kolmogorov–Smirnov and Levene’s tests, respectively.

For the non-cementation condition, adaptation parameters (AMD, MG, SA, AS, OS, and overall gap) were compared among the three crown materials (HS, VS, and CE) using one-way ANOVA followed by Tukey’s HSD post hoc test. For the cementation condition, the specimens previously evaluated under the non-cementation condition were returned to their respective material groups before random allocation into cement subgroups. Consequently, post-cementation data comprised six independent material–cement groups. Because CE could not be reliably evaluated after cementation owing to its radiopacity, only HS and VS were included in the post-cementation analyses. Overall gap and individual adaptation parameters (AMD, MG, SA, AS, and OS) were analyzed using two-way ANOVA with crown material and cement type as fixed factors, followed by Tukey’s HSD post hoc test. Statistical significance was set at *p* < 0.05.

## 3. Results

### 3.1. Non-Cementation Marginal and Internal Adaptation

**Figure 4:**
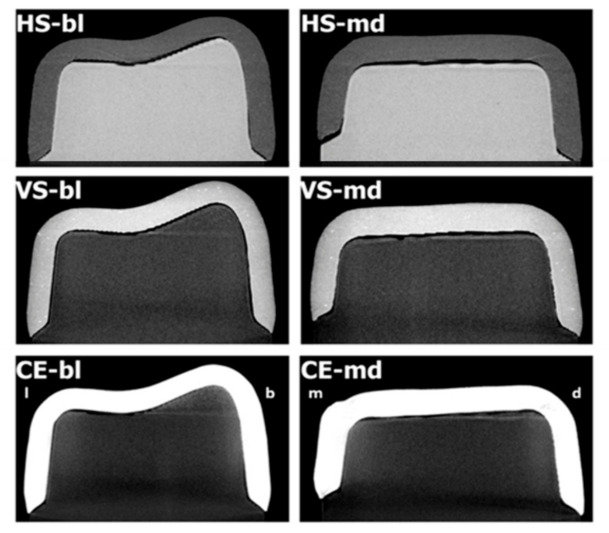
Representative micro-CT images of CRP crowns without cementation fabricated from HS (**top**), VS (**middle**), and CE (**bottom**) in bucco-lingual (bl) and mesio-distal (md) views. b = buccal; l = lingual; m = mesial; d = distal; HS = Hassawat-01, VS = VarseoSmile Crown Plus^®^; CE = Cerasmart^®^ 270.

Overall gap values prior to cementation (Table 2, Figure 4) revealed significant differences among the three CRP materials (*p* < 0.05). HS (94.12 ± 57.16 µm) and VS (97.71 ± 52.16 µm) showed significantly lower overall values than CE (108.41 ± 69.43 µm) (*p* < 0.05). No significant difference was observed between HS and VS (*p* > 0.05).

#### 3.1.1. Marginal Adaptation

As shown in Table 3, significant differences were observed in AMD among the materials (*p* < 0.05). HS exhibited the highest AMD (160.94 ± 20.67 µm), followed by CE (147.46 ± 42.48 µm), while VS showed the lowest value (120.18 ± 20.05 µm). HS exhibited significantly higher AMD than VS (*p* < 0.05), whereas no significant difference was observed between HS and CE, or between CE and VS. For MG, no significant differences were observed among the materials (*p* > 0.05). HS exhibited the lowest MG value (64.82 ± 16.44 µm), followed by CE (71.82 ± 45.00 µm) and VS (73.77 ± 28.58 µm).

#### 3.1.2. Internal Adaptation

Regarding internal adaptation, HS demonstrated the smallest internal gap at the shoulder area (SA) (120.00 ± 16.79 µm), followed by VS (131.91 ± 20.28 µm) and CE (154.41 ± 36.85 µm), with significant differences observed among the materials (*p* < 0.05). For the axial space (AS), HS exhibited a significantly smaller gap (44.47 ± 1.67 µm) compared with both CE (62.12 ± 15.71 µm) and VS (60.11 ± 7.09 µm) (*p* < 0.05), whereas CE and VS showed comparable values (*p* > 0.05). For the occlusal space (OS), no significant differences were observed among the three materials, with mean values of 138.34 ± 25.85 µm for HS, 137.68 ± 30.63 µm for VS, and 153.54 ± 67.49 µm for CE (*p* > 0.05) (Table 3).

### 3.2. Post-Cementation Adaptation

As shown in Table 2, overall gap values increased after cementation in both HS and VS. For HS, the overall gap increased from 94.12 ± 57.16 µm to 222.59 ± 159.50 µm with Maxcem Elite^®^, 269.30 ± 188.89 µm with RelyX Unicem^®^, and 227.72 ± 182.38 µm with Ketac Cem^®^. For VS, corresponding values were 155.76 ± 104.13 µm, 141.45 ± 85.16 µm, and 134.60 ± 104.58 µm, representing mean increases of 58.04, 43.90, and 36.88 µm, respectively. Across all cement types, HS demonstrated larger post-cementation gap increases than VS.

**Figure 5:**
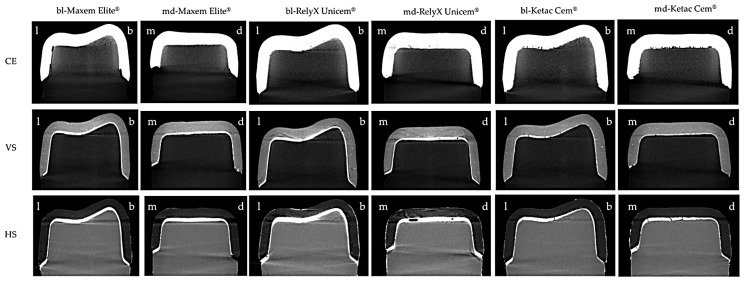
Representative micro-CT sectional images showing marginal and internal adaptation of CRP crowns after cementation. Images are presented for three materials: Hassawat-01 (HS), VarseoSmile Crown Plus^®^ (VS), and Cerasmart^®^ 270 (CE), luted with three different cements (Maxcem Elite^®^, RelyX Unicem^®^, and Ketac Cem^®^). b = buccal; l = lingual; m = mesial; d = distal.

Representative micro-CT images (Figure 5) illustrate cement distribution patterns after cementation. Post-cementation analysis was limited to HS and VS, as the radiopacity of CE overlapped with that of the luting cements, preventing reliable cement interface segmentation. In HS and VS specimens, the cement layer was clearly visible as a radiolucent band in both bucco-lingual and mesio-distal views. Resin-based cements (Maxcem Elite^®^ and RelyX Unicem^®^) showed more uniform and continuous cement layers with only isolated voids, whereas Ketac Cem^®^ exhibited irregular distribution with frequent voids and crack-like features in DLP-printed crowns. HS specimens demonstrated relatively larger internal gaps after cementation, particularly in the occlusal region, whereas VS showed more consistent adaptation patterns across sections.

#### 3.2.1. Marginal Adaptation

Post-cementation marginal adaptation values (AMD and MG) differed significantly between HS and VS across all cement types (*p* < 0.05) (Table 4). For HS, AMD values were 298.00 ± 76.27 µm with Maxcem Elite^®^, 360.09 ± 107.68 µm with RelyX Unicem^®^, and 262.32 ± 74.96 µm with Ketac Cem^®^. The corresponding MG values were 239.73 ± 120.67 µm, 324.84 ± 112.00 µm, and 287.77 ± 85.79 µm, respectively. Among the cements tested, Ketac Cem^®^ produced the lowest AMD for HS, while RelyX Unicem^®^ produced the highest. For VS, AMD values were 194.64 ± 67.63 µm with Maxcem Elite^®^, 125.39 ± 33.94 µm with RelyX Unicem^®^, and 180.50 ± 66.52 µm with Ketac Cem^®^. The corresponding MG values were 148.86 ± 73.59 µm, 90.73 ± 33.14 µm, and 128.05 ± 69.23 µm, respectively. RelyX Unicem^®^ produced the lowest AMD and MG for VS. Across all cement types, HS exhibited greater AMD and MG values than VS. A significant interaction between material and cement type was observed for both AMD and MG (*p* < 0.05).

#### 3.2.2. Internal Adaptation

Internal adaptation after cementation differed significantly between HS and VS for SA and OS (*p* < 0.001), whereas AS was significantly influenced by cement type rather than material (*p* < 0.05) (Table 4).

At the SA, HS exhibited larger gaps than VS for all cement types (*p* < 0.05). SA values for HS were 279.73 ± 103.34 µm, 317.86 ± 107.44 µm, and 283.41 ± 79.03 µm with Maxcem Elite^®^, RelyX Unicem^®^, and Ketac Cem^®^, respectively. VS showed smaller SA gaps of 212.91 ± 68.57 µm, 153.91 ± 33.53 µm, and 168.00 ± 76.04 µm with the corresponding cements.

For the AS, no significant difference was observed between HS and VS (*p* > 0.05); however, cement type significantly affected AS values (*p* < 0.05). HS showed AS values of 92.74 ± 11.77 µm, 93.50 ± 13.70 µm, and 67.03 ± 8.43 µm, while VS showed 78.92 ± 17.06 µm, 95.12 ± 20.77 µm, and 63.53 ± 7.05 µm with Maxcem Elite^®^, RelyX Unicem^®^, and Ketac Cem^®^, respectively. Ketac Cem^®^ produced significantly smaller AS values than both Maxcem Elite^®^ and RelyX Unicem^®^ in both materials (*p* < 0.05).

For the occlusal space (OS), HS exhibited markedly larger gaps than VS across all cement types (*p* < 0.05). OS values for HS were 336.98 ± 110.47 µm, 419.72 ± 124.35 µm, and 346.80 ± 128.70 µm, compared with 226.43 ± 80.48 µm, 237.09 ± 49.69 µm, and 207.55 ± 98.09 µm for VS, with Maxcem Elite^®^, RelyX Unicem^®^, and Ketac Cem^®^, respectively. No significant effect of cement type or interaction was observed for OS (*p* > 0.05).

## 4. Discussion

Prior to cementation, the additively manufactured HS and VS crowns demonstrated significantly smaller overall marginal and internal gaps than the milled CE crowns, with no significant difference between the two printed groups (Table 2). Location-specific analysis confirmed that HS demonstrated the smallest internal gaps particularly at the shoulder and axial regions, VS exhibited more favorable marginal adaptation, and CE consistently showed larger internal gaps across all regions (Table 3). After cementation, both HS and VS showed significant increases in overall, marginal and internal gaps, indicating that the cementation process substantially altered restoration adaptation. The magnitude and pattern of these changes varied with both crown material and cement type, suggesting a strong crown material–cement interaction rather than a uniform hydraulic effect. Collectively, these findings led to rejection of all three null hypotheses.

The superior non-cementation internal adaptation of the additively manufactured crowns is consistent with previous reports describing higher dimensional accuracy of DLP fabrication, particularly in complex internal geometries [21,22,24,46]. The layer-by-layer photopolymerization enables accurate reproduction of fine anatomical details that are geometrically inaccessible to rotary milling tools [57,58]. In contrast, subtractive milling is constrained by bur diameter and tool-path geometry, leading to unavoidable over-milling when intaglio curvature is smaller than the bur radius [18,21]. This difference persisted despite systematic bur replacement every 17 crowns, suggesting that the improved internal adaptation of DLP-printed crowns is inherent to the additive manufacturing process rather than a consequence of suboptimal milling parameters. These findings are consistent with previous reports that both fabrication methods can achieve clinically acceptable gap dimensions, although internal gap distribution differs according to manufacturing parameters such as printing angulation [1,24]. In addition, Yassine et al. and Herguner et al. reported that DLP-printed crowns demonstrated superior internal fit but inferior marginal adaptation compared with milled counterparts, with outcomes varying according to resin material and printer system [26,59].

All preparations incorporated a convergence angle of 6° per axial wall, and the low non-cementation axial space (AS) values in both HS and VS indicated that DLP-printed CRP crowns can achieve very close axial adaptation under conservative taper conditions. Previous studies on the effects of convergence angle on crown adaptation remain inconsistent, with some reporting improved adaptation at larger tapers [60,61,62], while others found no significant effect [63]. In HS, the extremely tight axial conformity likely restricted internal volume available for cement escape during seating. Clinically, minor refinement of the tooth abutment with fine-grit finishing burs to increase convergence angle may help reduce hydraulic resistance when DLP-printed restorations exhibit excessively tight adaptation during try-in, although this approach requires further experimental validation.

Post-cementation evaluation of CE crowns was not feasible because the radiopacity of the milled resin nanoceramic closely matched that of the luting cements, preventing reliable cement interface segmentation in micro-CT reconstructions. Attempts to optimize tube voltage to improve interfacial contrast for CE resulted in substantially reduced image quality for HS specimens. Because HS represents the novel material under primary investigation, scanning parameters were optimized for HS visualization, precluding quantitative post-cementation analysis of CE. This material-dependent limitation of single-energy micro-CT has been noted in the broader restoration fit literature [64]. Future approaches incorporating radiopaque contrast agents, dual-energy CT, or radiopaque modifiers in restorative materials may enable reliable post-cementation visualization across a wider range of CRP materials [64,65].

Cementation markedly influenced marginal and internal adaptation, even when non-cementation fit appeared favorable. HS demonstrated the closest non-cementation fit, particularly at the shoulder (SA: 120.00 µm) and axial regions (AS: 44.47 µm), but exhibited the greatest increases in post-cementation gaps. The restricted internal volume of HS most likely impeded cement escape during seating, generating greater hydraulic pressure and subsequent marginal opening [38,66]. In contrast, VS crowns, with moderate non-cementation internal relief (SA: 131.91; AS: 60.11 µm), showed only moderate increases in gap after cementation across all cement types. This aligns with the hydrodynamic model of Grajower and Lewinstein [38], where intracoronal pressure depends on internal volume, cement viscosity, and seating velocity. Adequate internal relief allows cement to escape, minimizes hydraulic back-pressure, and improves seating completeness. These findings suggest that a uniform cement space setting of 50 µm may be insufficient for highly accurate DLP-printed CRP crowns such as HS. Previous studies have recommended cement spaces of 60–110 µm to balance adaptation and hydraulic resistance [27,32,67,68,69]; however, the optimal cement space for CRP materials remains to be determined.

The effect of luting cement on post-cementation adaptation differed substantially between HS and VS, indicating that cement behavior was influenced by both intaglio geometry and cement rheology. In HS, post-cementation marginal gaps ranked as follows: Maxcem Elite^®^ < Ketac Cem^®^ < RelyX Unicem^®^, whereas AMD ranked as: Ketac Cem^®^ < Maxcem Elite^®^ < RelyX Unicem^®^ (Table 4). This pattern suggests that when internal relief is severely restricted, cement viscosity becomes a dominant determinant of seating behavior. During the active seating phase, higher-viscosity cements generate greater hydraulic resistance within confined internal spaces [38,70,71]. RelyX Unicem^®^, with high filler loading (~72 wt.%) and small filler particles (<9.5 µm) [72], exhibits the highest viscosity among the tested cements, potentially explaining the largest marginal discrepancies observed in HS. In contrast, Maxcem Elite^®^, with slightly lower filler content (~69wt.%) and comparable particle size [73], has lower viscosity, thereby permitting more efficient cement escape and resulting in smaller gaps. Ketac Cem^®^, despite its high elastic modulus, has lower initial viscosity as a conventional glass-ionomer cement [55], facilitating cement flow during seating [38,71]. These observations agree with previous reports showing increased marginal misfit with higher-viscosity cements [71].

In VS, RelyX Unicem^®^ produced the smallest marginal discrepancies, while Maxcem Elite^®^ and Ketac Cem^®^ showed larger marginal openings (Table 4). Although all cements have film thickness within ISO limits (RelyX Unicem^®^: 19.4 ± 4.8 µm, Maxcem Elite^®^: 37.6 ± 8.7 µm, and Ketac Cem^®^: 19.5 µm) [70,74,75], Ketac Cem^®^ did not consistently improve marginal adaptation despite its relatively low film thickness, indicating that film thickness alone does not determine seating behavior [76]. The slightly greater internal relief of VS may have functioned as a pressure-relief reservoir during seating [38], reducing sensitivity to cement viscosity. Under these conditions, the rheological properties of RelyX Unicem^®^ may have provided a favorable balance between flowability and maintenance of a continuous cement layer [70], consistent with previous micro-CT studies reporting superior marginal adaptation with RelyX Unicem^®^ compared with other resin-based cements [77].

Following seating, the elastic modulus of the set cement may further influence the final marginal gap. Li and White [78] demonstrated that resistance to plastic deformation contributes to resistance against marginal gap formation, while finite element analyses have shown that cement modulus affects stress distribution within the crown–cement–tooth complex [79]. Accordingly, a stiffer cement layer may reduce elastic rebound after removal of the seating force, resulting in a smaller final marginal gap, although this mechanism remains hypothetical [78,79]. In dual- or chemical-cure luting cements, modulus development during polymerization may also affect seating stability if continuous pressure is not maintained until complete hardening, potentially influencing plastic deformation behavior and marginal adaptation; however, this remains speculative. In the present study, cement moduli differed substantially: Ketac Cem^®^ (~20 GPa) > RelyX Unicem^®^ (~13 GPa) > Maxcem Elite^®^ (~4 GPa) [15]. The high modulus of Ketac Cem^®^ may therefore have partially compensated for the hydraulic-induced displacement, producing a smaller marginal gap than RelyX Unicem^®^ in HS. Nevertheless, Maxcem Elite^®^ produced the smallest marginal gap overall in HS, possibly because its lower viscosity improved cement escape and seating efficiency. Collectively, these findings suggest that viscosity-driven hydraulic effects during seating may have a greater influence on marginal adaptation in HS than modulus-related rebound effects.

The contrasting behaviors of HS and VS further support this interpretation. Compared with HS, the greater internal relief of VS appears to have reduced sensitivity to cement viscosity by providing additional escape volume during seating [38]. Consequently, cement rheology had less influence on seating completeness in VS, whereas the restricted internal space in HS amplified viscosity-related hydraulic effects and altered the relative cement performance ranking. Clinically, these findings suggest that cement selection optimized for one DLP-printed CRP material may not be directly transferable to another material with different internal geometry, even when fabricated using the same printing technology. Future finite element studies are warranted to independently evaluate the relative contributions of cement viscosity and elastic modulus within geometrically accurate CRP crown–die models.

Qualitative micro-CT observations revealed differences in cement morphology among the groups (Figure 5). Resin-based cements (Maxcem Elite^®^ and RelyX Unicem^®^) showed more homogeneous and continuous cement interfaces, whereas Ketac Cem^®^ exhibited irregular distribution with voids and crack-like features, particularly in DLP-printed crowns (HS and VS). These findings may partly explain the lower fracture resistance and distinct failure patterns previously reported for glass-ionomer cement in these materials [15]. Although HS exhibited larger post-cementation marginal gaps than VS, it predominantly showed superficial cracking without catastrophic fracture. The thicker cement layer associated with larger gaps may amplify the mechanical influence of luting cements with different elastic moduli on stress distribution within the crown–cement complex. In addition, the alumina reinforcement in HS may contribute to fracture resistance, while the urethane acrylate-based matrix is expected to contribute greater toughness than Bis-GMA–dominant systems. Collectively, these findings suggest that marginal adaptation alone does not predict fracture performance, and that cement properties and material-specific matrix–filler characteristics should also be considered when evaluating CRP crown systems. Interestingly, our previous study found no clear correlation between marginal discrepancy and fracture resistance among HS, VS, and CE crowns with different occlusal thicknesses [24]. However, that study used only a single resin cement and evaluated marginal adaptation only before cementation, without assessing internal gap parameters, which may directly influence stress distribution and fracture behavior more than marginal discrepancy alone.

From a clinical perspective, most post-cementation marginal values exceeded the traditionally cited 120 µm threshold, which was originally established for zinc phosphate cement with relatively high solubility [17]. Because resin-based cements exhibit significantly lower solubility and stronger adhesive bonding [80,81,82], marginal discrepancies moderately above this threshold may still be clinically acceptable when internal adaptation and interfacial sealing are adequate. In the present study, HS crowns consistently exceeded 200 µm across all cement types, surpassing both the clinically acceptable threshold of 120 µm and the 150 µm level associated with increased cement dissolution [17], whereas VS crowns luted with RelyX Unicem^®^ achieved MG values within the clinically acceptable range (90.73 µm). These findings suggest that HS may require a larger cement space and lower-viscosity cements to reduce hydraulic resistance and improve seating adaptation [27,32,67,68,69], whereas a 50 µm cement space setting combined with RelyX Unicem^®^ is suitable for VS crowns. Nevertheless, these recommendations should be validated through controlled experimental and clinical studies.

Several limitations should be acknowledged. The 10 µm/voxel micro-CT resolution imposes a minimum detectable gap of approximately 40 µm, which may limit precision in regions with very small internal spaces, particularly in HS. In addition, this in vitro study used standardized resin dies that cannot fully reproduce clinical variability, including differences in dentin properties, tooth preparation geometry, and other intraoral factors. No universally accepted standard currently exists for the number and distribution of measurement points in adaptation analyses [44,45]; although Groten et al. [83] suggested that 50 measurements may be sufficient, this study adopted a 160-point protocol based on previously established methods [46], which may limit direct inter-study comparisons. Furthermore, post-cementation analysis of CE was not feasible due to radiopacity overlap with the luting cements, as noted above. Clinically, crowns exhibiting incomplete seating or increased marginal and internal gaps may require additional intra-oral occlusal adjustment and re-polishing.

Future studies should extend the present findings by addressing several complementary research questions. In the short term, in vitro studies should optimize cement space settings for alumina-reinforced DLP-printed CRP materials, evaluate the influence of total occlusal convergence on post-cementation adaptation, quantify three-dimensional cement thickness and void distribution using volumetric micro-CT analysis, and further investigate the interactions among cement rheology, filler characteristics, and seating behavior. Future investigations should also clarify the relationship between post-cementation adaptation and the long-term mechanical performance of CRP restorations under clinically relevant aging conditions. In the medium term, finite element analysis combined with thermomechanical aging and fatigue loading would provide further insight into stress distribution within the crown–cement–tooth complex. Ultimately, long-term clinical studies are required to validate the present in vitro findings under functional conditions.

## 5. Conclusions

Within the limitations of this study, the following conclusions were drawn:

- Before cementation, HS showed the best internal adaptation (overall gap 94.12 ± 57.16 µm) but the highest marginal discrepancy; VS showed the best marginal adaptation; and CE showed the largest internal gaps.
- After cementation, HS exhibited the greatest increase in marginal and internal discrepancies across all cement types, suggesting substantial hydraulic resistance during seating.
- VS luted with RelyX Unicem^®^ achieved the most favorable post-cementation adaptation and was the only combination within the clinically acceptable marginal gap range.
- Luting cement type significantly influenced post-cementation adaptation, with cement effects being more pronounced in HS than in VS, underscoring the importance of material–cement compatibility in clinical crown selection.

## Figures and Tables

**Table 1:** Details of the materials used in this research [15].

Group	Product	Manufacturer	Composition	Lot. No.
**DLP-Printing**	Hassawat-01 (HS)	Faculty of Science, Chulalongkorn University, Thailand	Urethane acrylate, 2-Phenoxyethyl acrylate, Tripropyleneglycol diacrylate, Trimethylolpropane triacrylate, Trimethyl benzoyl diphenyl phosphine oxide (61.5 wt.% total resin), and Aluminum oxide 0.7 µm 38.5 wt.%	N/A
	VarseoSmile Crown Plus^®^ (VS)	Bego, Bremen, Germany	Esterification products of 4.4′-isopropylidiphenol, ethoxylated and 2-methylprop-2enoic acid. Silanized dental glass, methyl benzoylformate, diphenyl (2,4,6-trimethylbenzoyl) phosphine oxide. Total content of inorganic fillers (particle size 0.7 μm) is 30–50 wt.%	601817
**Milling**	Cerasmart^®^ 270 (CE)	GC, Tokyo, Japan	Bis-MEPP, UDMA, dimethacrylate, silica (20 nm), and barium glass (300 nm) 71 wt.%	2108056
**Luting agent materials**	Maxcem Elite^®^	SDS Kerr, Orange, CA, USA	Glyceroldimethacrylate dihydrogen phosphate (GPDM) hydroxyethylmethacrylat (HEMA), 4-methoxyphenol (MEHQ), cumolhydroperoxid (CHPO), methacrylate ester monomers, titanium dioxide, pigments	A159983
	RelyX Unicem^®^	3M ESPE, Saint Paul, USA	Powder: glass fillers, silica, calcium hydroxide, self-curing initiators, pigments, light-curing initiators, substituted pyrimidine, peroxy compound. Liquid: methacrylated phosphoric esters, dimethacrylates, acetate, stabilizers, self- curing initiators, light- curing initiators	10684647
	Ketac Cem^®^	3M ESPE, Saint Paul, USA	Powder: Glass powder Liquid: Polycarboxylic acid, Pigments Tartaric acid, Water, Conservation agents	10605282
**Silane Coupling Agent**	RelyX Ceramic Primer^®^	3M ESPE, Saint Paul, USA	Ethyl alcohol, water, 3-methacryloxypropyltrimethoxysilane	NF44358
**Resin die**	Rigid 10K Resin	Formlabs Inc., USA	Highly glass-filled resins-based methacrylate	N/A

N/A = not applicable.

**Table 2:** Mean ± SD values of overall marginal and internal gap measurement.

Crown Types	Non-Cementation (µm) ± SD	Cement Types (µm) ± SD		
	(n = 11)	Maxcem Elite^®^ (n = 11)	RelyX Unicem^®^ (n = 11)	Ketac Cem^®^ (n = 11)
HS	94.12 ^b^ ± 57.16	222.59 ^Aa^ ± 159.50	269.30 ^Ba^ ± 188.89	227.72 ^Aa^ ± 182.38
VS	97.71 ^b^ ± 52.16	155.76 ^Ab^ ± 104.13	141.45 ^Bb^ ± 85.16	134.60 ^Cb^ ± 104.58
CE	108.41 ^a^ ± 69.43	N/A	N/A	N/A

Mean values with different lowercase superscript letters within the same column and different uppercase superscript letters within the same row indicate significantly different (*p* ≤ 0.05). HS = Hassawat-01; CE = Cerasmart^®^ 270; VS = VarseoSmile Crown Plus^®^; N/A = not applicable; n = number of specimens per group.

**Table 3:** Non-cementation marginal and internal adaptation of ceramic-reinforced polymer crowns (mean ± SD).

Material	Marginal Gap		Internal Gap		
	AMD (µm)	MG (µm)	SA (µm)	AS (µm)	OS (µm)
HS	160.94 ± 20.67 ^a^	64.82 ± 16.44 ^a^	120.00 ± 16.79 ^b^	44.47 ± 1.67 ^b^	138.34 ± 25.85 ^a^
VS	120.18 ± 20.05 ^b^	73.77 ± 28.58 ^a^	131.91 ± 20.28 ^ab^	60.11 ± 7.09 ^a^	137.68 ± 30.63 ^a^
CE	147.46 ± 42.48 ^ab^	71.82 ± 45.00 ^a^	154.41 ± 36.85 ^a^	62.12 ± 15.71 ^a^	153.54 ± 67.49 ^a^

Values are presented as mean ± SD (n = 11 crowns per subgroup). Different superscript letters within the same column indicate significant differences (*p* ≤ 0.05). AMD = absolute marginal discrepancy; MG = marginal gap; SA = shoulder area; AS = axial space; OS = occlusal space.

**Table 4:** Post-cementation marginal and internal adaptation of DLP-printed ceramic-reinforced polymer crowns (mean ± SD).

Luting Cement	Material	Marginal Gap		Internal Gap		
		AMD (µm)	MG (µm)	SA (µm)	AS (µm)	OS (µm)
Maxcem Elite^®^	HS	298.00 ± 76.27 **^A^**	239.73 ± 120.67 **^A^**	279.73 ± 103.34 **^A^**	92.74 ± 11.77 **^A^**^a^	336.98 ± 110.47 **^A^**
	VS	194.64 ± 67.63 **^B^**	148.86 ± 73.59 **^B^**	212.91 ± 68.57 **^B^**	78.92 ± 17.06 **^A^**^a^	226.43 ± 80.48 **^B^**
RelyX Unicem^®^	HS	360.09 ± 107.68 **^A^**	324.84 ± 112.00 **^A^**	317.86 ± 107.44 **^A^**	93.50 ± 13.70**^A^**^a^	419.72 ± 124.35 **^A^**
	VS	125.39 ± 33.94 **^B^**	90.73 ± 33.14 **^B^**	153.91 ± 33.53 **^B^**	95.12 ± 20.77**^A^**^a^	237.09 ± 49.69 **^B^**
Ketac Cem^®^	HS	262.32 ± 74.96 **^A^**	287.77 ± 85.79 **^A^**	283.41 ± 79.03 **^A^**	67.03 ± 8.43 **^Ab^**	346.80 ± 128.70 **^A^**
	VS	180.50 ± 66.52 **^B^**	128.05 ± 69.23 **^B^**	168.00 ± 76.04 **^B^**	63.53 ± 7.05**^Ab^**	207.55 ± 98.09 **^B^**

Values are mean ± SD (n = 11 specimens per subgroup). Different uppercase superscript letters within the same column indicate significant differences between materials (HS vs. VS). Different lowercase superscript letters indicate significant differences among cement types *p* < 0.05. AMD = absolute marginal discrepancy; MG = marginal gap; SA = shoulder area; AS = axial space; OS = occlusal space. HS = Hassawat-01; VS = VarseoSmile Crown Plus^®^.

## Data Availability

The original contributions presented in this study are included in the article. Further inquiries can be directed to the corresponding author.

## Abbreviations

AMD	Absolute marginal discrepancy
ANOVA	Analysis of variance
AS	Axial space
Bis-GMA	Bisphenol A-glycidyl methacrylate
CAD/CAM	Computer-aided design/Computer-aided manufacturing
CE	Cerasmart^®^ 270
CRP	Ceramic-reinforced polymer
DLP	Digital light processing
GPDM	Glycerol dimethacrylate dihydrogen phosphate
HEMA	Hydroxyethyl methacrylate
HS	Hassawat-01
HSD	Honestly significant difference
ICC	Intraclass correlation coefficient
ISO	International Organization for Standardization
LED	Light-emitting diode
MG	Marginal gap
Micro-CT	Micro-computed tomography
OS	Occlusal space
PICN	Polymer-infiltrated ceramic network
RNC	Resin nanoceramic
SA	Shoulder area
SD	Standard deviation
SLA	Stereolithography
TEGDMA	Triethylene glycol dimethacrylate
UA	Urethane acrylate
UDMA	Urethane dimethacrylate
VS	VarseoSmile Crown Plus^®^
